# Data on draft genomes and transcriptomes from females and males of the flour moth, *Ephestia kuehniella*

**DOI:** 10.1016/j.dib.2022.108140

**Published:** 2022-04-07

**Authors:** Axel Künstner, Hauke Busch, Enno Hartmann, Walther Traut

**Affiliations:** a Medical Systems Biology Group, Lübeck Institute for Experimental Dermatology, University of Lübeck, Ratzeburger Allee 160, Lübeck 23562, Germany; b Institut für Biologie, Zentrum für Medizinische Struktur- und Zellbiologie, Universität zu Lübeck, Ratzeburger Allee 160, Lübeck 23562, Germany

**Keywords:** Female and male genomes, Female and male transcriptomes, Lepidoptera, *de novo* assembly, Heterozygosity

## Abstract

We present genomes and pupal transcriptomes of the Mediterranean flour moth, *Ephestia kuehniella*. The moth is a world-wide storage pest as well as a laboratory species with a considerable background in developmental biology, genetics, and cytogenetics. The sequence data were derived from a highly inbred laboratory strain and, hence, display very little heterozygosity. Female and male genomes and transcriptomes are represented separately in two sets each of raw and assembled sequence data. They are designed as a basis to develop new strategies in pest control, to elucidate the molecular adaptation for its peculiar lifestyle, and for research on sex chromosome structure, sex determination and sex-specific gene activity. For a test, all genes known or suspected to have a role in sex determination were extracted from the data. Raw sequencing data and assemblies are available at European Nucleotide Archive under accession number PRJEB49052.

## Specifications Table

**Table:** 

Subject	Entomology and insect science
Specific subject area	Insects, Lepidoptera, Genomics, Transcriptomics
Type of data	Raw data from DNA and RNA-sequencing of females and males (fastq files) Genome assembly (fasta files) Transcriptome assembly (fasta files) Table with ENA accession numbers and Genbank IDs Table showing summary statistics of the assemblies
How the data were acquired	Illumina HiSeq 2500 sequencing platform, paired-end sequencing data for DNA and RNA, mate pairs with 8 kb insert size for DNA
Data format	Raw data Analyzed data
Parameters of data collection	Data collection contains raw genome and transcriptome data for two different datasets (referred to as Mainz and Novogene) of the laboratory strain L of *E. kuehniella*. Additionally, transcriptome assemblies of both datasets and a genome assembly of the dataset Mainz are available.
Description of data collection	Total RNA and total DNA from females and males was extracted from mid-stage pupae of laboratory strain L of *E. kuehniella* (Mainz: one female and one male each; dataset Novogene: 5 females and 5 males each and pooled by sex) and subjected to HiSeq Illumina paired-end (Mainz: 2 × 250 bp; Novogene: 2 × 150 bp) and mate-pair (Mainz only, 8 kb insert size, 2 × 250 bp) sequencing.
Data source location	• Institution: University of Lübeck • City/Town/Region: Lübeck • Country: Germany
Data accessibility	The raw sequence reads, and the assemblies can be obtained through ENA study accession number PRJEB49052. Gene sequences for sex determining genes were submitted to Genbank. Repository name: European Nucleotide Archive (ENA) Data identification number: PRJEB49052 Direct URL to data: https://www.ebi.ac.uk/ena/browser/view/PRJEB49052

## Value of the Data

• The source of the genomes and transcriptomes, *E. kuehniella*, is a storage pest with world-wide distribution. It is also a favorable laboratory species and has a rich background in developmental biology, genetics, and cytogenetics.
• Researchers developing new molecular strategies of pest control will benefit from the data as well as those interested in insect phylogeny, genetic adaptation for the peculiar lifestyle of the species and its sex determination, sex chromosome content and sex-specific expression of genes.
• The female and male genomes and transcriptomes are from a highly inbred line and have a very low level of heterozygosity. This makes them especially valuable for female-versus-male comparisons. The developmental stage, mid-pupa, is a stage when genes involved in morphogenesis and sex-differentiation are supposed to be active.

## 1 Data Description

The dataset contains a draft genome and draft pupal transcriptome assembly, separately from females and males of the Mediterranean flour moth, *E. kuehniella* (Lepidoptera, Pyralidae), besides two sets of raw sequencing data referred to as ‘Mainz’ and ‘Novogene’. For dataset ‘Mainz’, data from a highly inbred line was collected from a single female and a single male individual for the transcriptome assembly and from two female and two male individuals for the genome assembly. RNA libraries were submitted to paired-end Illumina sequencing and DNA libraries were sequenced using paired-end and 8 kb mate-pair Illumina sequencing technology (dataset ‘Mainz’). The raw sequencing data and the assemblies can be obtained from ENA study accession number PRJEB49052 (accession numbers for genome assemblies derived from the ‘Mainz’ dataset: female ERS8464940, male ERS8464941; accession numbers for transcriptome assemblies: female ERS8464942, male ERS8464943; more details are given in Table 1). A second set of raw data (dataset ‘Novogene’) was obtained by pooling 5 females and 5 males separately of the same inbred *E. kuehniella* strain and can be obtained from the same study accession number. RNA and DNA libraries from this dataset were sequenced using paired-end sequencing and the RNA-seq data was used to perform a second transcriptome assembly for each sex (accession numbers for transcriptome assemblies derived from the ‘Novogene’ dataset: female ERS8464945, male ERS8464946).

**Table 1:** Accession numbers for the European Nucleotide Archive (ENA) for the sequencing data and Genbank IDs for *E. kuehniella* orthologs genes known or suspected to have a role in its sex determination.

Repository	ID	Dataset
**ENA**	ERS8464763	Female genome Mainz raw data
	ERS8464764	Female transcriptome Mainz raw data
	ERS8464765	Female genome Mainz raw data (8 kb mate pairs)
	ERS8464766	Male genome Mainz raw data
	ERS8464767	Male transcriptome Mainz raw data
	ERS8464768	Male genome Mainz raw data (8 kb mate pairs)
	ERS8464769	Female genome Novogene raw data
	ERS8464770	Female genome Novogene raw data
	ERS8464771	Female transcriptome Novogene raw data
	ERS8464772	Male genome Novogene raw data
	ERS8464773	Male genome Novogene raw data
	ERS8464774	Female transcriptome Novogene raw data
	ERS8464940	Female genome assembly Mainz
	ERS8464941	Male genome assembly Mainz
	ERS8464942	Female transcriptome assembly Mainz
	ERS8464943	Male transcriptome assembly Mainz
	ERS8464944	Combined female/male transcriptome assembly Novogene
	ERS8464945	Female transcriptome assembly Novogene
	ERS8464946	Male transcriptome assembly Novogene

**Genbank**	OU228360	E. kuehniella mRNA for Ekdsx f1 (female splice variant 1)
	OU228361	E. kuehniella mRNA for Ekdsx f2 (female splice variant 2)
	OU228362	E. kuehniella mRNA for Ekdsx m1(male splice variant 1)
	OU228363	E. kuehniella mRNA for fruitless (Ekfruitless)
	OU228364	E. kuehniella mRNA for heatshock protein 70 (EkHSP70)
	OU228365	E. kuehniella mRNA for IGF-II mRNA binding protein (EkIMP)
	OU228366	E. kuehniella mRNA for P-element somatic inhibitor (EkPSI)
	OU228367	E. kuehniella mRNA for Sex lethal (EkSxl)
	OU228368	E. kuehniella mRNA for transformer-2 (EkTra2)

Genome sizes for the haploid female and male genomes were estimated using the ‘Novogene’ data. Estimates based on a *kmer* approach were 363Mb (megabases) for the haploid female genome and 365Mb for the male genome. This is significantly less than 440Mb, the value determined by flow cytometry and confirmed by Feulgen cytometry [1]. Assembled genomes were 357Mb (female) and 354Mb (male) with an N50 of 11,860 bp (female) and 12,636 bp (male). The longest contigs were ∼197 kb in the female genome assembly and ∼426 kb in the male assembly, respectively. GC content was very similar between the two sexes (∼36%). Further assembly details are shown in Table 2. The completeness of the genome and transcriptome assemblies was assessed using BUSCO with the lepidoptera-odb10 lineage dataset (Table 2). The ‘Novogene’ dataset was used to estimate heterozygosity. As expected from a highly inbred line, heterozygosity was very low. For the female genome, heterozygosity was estimated between 0.152 and 0.156% and for the male assembly the estimated heterozygosity was between 0.034 and 0.037%. The higher estimate of heterozygosity in females is probably due to the fact that females are the heterogametic sex in *E. kuehniella* and have WZ sex chromosomes while males are homogametic with a ZZ sex chromosome pair.

**Table 2:** Assembly statistics for the genome and transcriptome assembly and results from benchmarking universal single-copy orthologs (BUSCO) analysis against lepidoptera-odb10 as reference dataset for genome and transcriptome completeness. Percentage of genes per assembly from BUSCO analysis are shown for complete single copy and duplicated genes as well as for fragmented genes (5286 genes in total).

		Genome Mainz		Transcriptome Mainz		Transcriptome Novogene	
Features		Female	Male	Female	Male	Female	Male
**Assembly**	Size (bp)	357,446,945	353,955,619	61,075,989	68,091,461	144,998,424	157,359,019
	Sequences	90,999	90,445	87,516	101,822	141,949	150,130
	N50 (bp)	11,860	12,636	10,905	13,268	2271	2335
	L50	7830	7172	1253	1127	16,542	17,474
	N90 (bp)	1662	1636	268	264	355	364
	L90	41,028	39,236	61,107	72,169	88,955	93,588
	Longest contig (bp)	197,352	425,840	24,754	36,418	49,425	49,499
	Gaps (runs of Ns)	34,083	25,950	2581	2965	0	0
	Number N	31,356,943	30,452,154	22,651	26,965	0	0
	GC (%)	35.97	35.96	38.43	38.07	41.27	41.11

**BUSCO**	Complete	4352	4477	3998	3972	4790	4956
	Complete: single copy	4313 (81.6%)	4440 (84.0%)	3961 (74.9%)	3923 (74.2%)	2875 (54.4%)	2872 (54.3%)
	Complete: duplicated	39 (0.7%)	37 (0.7%)	37 (0.7%)	49 (0.9%)	1915 (36.2%)	2084 (39.4%)
	Fragmented	528 (10.0%)	430 (8.1%)	406 (7.7%)	409 (7.7%)	79 (1.5%)	52 (1.0%)
	Missing	406 (7.7%)	379 (7.2%)	882 (16.7%)	905 (17.1%)	417 (7.9%)	278 (5.3%)

For a test of the data set, we searched for the *E. kuehniella* orthologs of all genes known or suspected to have a role in its sex determination. *EkMasc* and *EkMascB*, the orthologues of *Masculinizer* (*Masc*) from *Bombyx mori* were recently described to produce the primary signal of the sex determining cascade in *E. kuehniella* [2]. Our assemblies allowed us to extract these and *Ekdsx, EkPSI, EkIMP, EkTra2, EkSxl, EkHSP70,* as well as the sex-specific splice variants of *Ekdsx* (GenBank accession numbers: OU228360–OU228368; see Table 1 for details).

## 2 Experimental Design, Materials and Methods

### 2.1 Sample collection

*E. kuehniella* strain L has been kept in laboratory cultures for more than 80 years. For sequencing, female and male mid-stage pupae were selected. One female and one male pupa each were used in paired-end and 8 kb mate-pair genome sequencing as well as in paired end transcriptome sequencing for data set 'Mainz'. The ‘Novogene’ data set was derived from mid-stage pupae for RNA and DNA sequencing (five females and males each, which were pooled by sex for sequencing).

### 2.2 Library preparation and sequencing

For data set `Mainz', DNA and RNA extraction and sequencing was performed by StarSeq (Mainz, Germany) and for the 'Novogene' by Novogene (Hongkong, China). Short-read libraries were prepared using the TruSeq Library Preparation Kit, and sequencing was performed using the Illumina HiSeq 2500 system. For dataset ‘Mainz’ sequences were labeled with barcodes (female: paired-end genome CGAGGCTG/CAGCCTCG, paired-end transcriptome TCCGCGAA/TTCGCGGA, mate-pairs ACAGTGAT; male: paired-end genome CGTACTAG, paired-end transcriptome TCTCGCGC, mate-pairs GCCAATAT). For dataset ‘Novogene’ the barcodes were removed by Novogene. Libraries from ‘Mainz’ were sequenced using 2 × 250 bp reads and ‘Novogene’ was sequenced using 2 × 150 bp.

### 2.3 Genome and transcriptome assembly

Raw sequencing reads from dataset ‘Mainz’ were quality checked and adaptor sequences were removed. For both sexes, a *de novo* genome assembly was performed using CLC Assembly Cell v4.0 (QIAGEN Digital Insights, Redwood City, USA) applying a *deBruijn* graph model (parameter settings: bubblesize = 300, kdef which refers to default kmer size). Transcriptome assemblies for dataset `Novogene' were performed using Trinity [3] (v2.8.4) with the following command line:

Trinity –seqType fq –max_memory 180 G –left FORWARD.fastq –right BACKWARD.fastq –CPU 24 –trimmomatic –jaccard_clip –full_cleanup –output assembly_output

Quality assessment of genome and transcriptome assemblies was done applying BUSCO [4] (v4.0.4) using the lepidoptera-odb10 lineage dataset (creation date 2020–08–05) with no additional third-party components.

### 2.4 Data analysis

Genome size, and heterozygosity were estimated using a kmer-based approach as implemented in jellyfish [5] (*kmer* size 27) and resulting histograms were uploaded to GenomeScope [6] (http://qb.cshl.edu/genomescope) to perform the analysis.

## CRediT authorship contribution statement

**Axel Künstner:** Data curation, Investigation, Writing – original draft, Writing – review & editing. **Hauke Busch:** Writing – review & editing. **Enno Hartmann:** Writing – review & editing. **Walther Traut:** Conceptualization, Resources, Investigation, Data curation, Writing – original draft, Writing – review & editing.

## Declaration of Competing Interest

The authors declare that they have no known competing financial interests or personal relationships that could have appeared to influence the work reported in this paper.

## Data Availability

Sequencing read data and assemblies for Ephestia kuehniella draft genomes and transcriptomes (Original data) (European Nucleotide Archive (ENA)).
